# Headliners: Chemical Exposures and Childhood Leukemia: Parental Chemical Exposures and *ras* Mutations in Children

**Published:** 2004-10

**Authors:** Jerry Phelps

A variety of carcinogens have been shown to induce *ras* mutations in animal and human tumor models, and *ras* proto-oncogene mutations have been implicated in the development of many malignancies including pancreatic and breast cancers. However, few data exist associating parental exposures and *ras* mutations in their children. Now a team including NIEHS grantee Leslie L. Robison of the University of Minnesota report that parents’ chemical exposures may be associated with distinct *ras* mutations in their children with acute lymphoblastic leukemia (ALL).

This study used data from a large case–control study of childhood ALL conducted by the Children’s Oncology Group in Southern California. DNA samples from the study children were examined for *ras* mutations. A total of 127 out of 837 ALL cases exhibited *ras* mutations in the K- or N-*ras* genes. Earlier studies have reported a 5–20% frequency of *ras* mutations among patients with ALL.

A number of parental chemical exposures were associated with significantly increased risks for *ras* mutation in the children. Use of drugs such as marijuana, LSD, and cocaine was associated with increased risk of N-*ras* mutation (three-fold higher risk for maternal use and two-fold higher risk for paternal use). Paternal use of amphetamines or diet pills was associated with a four-fold increase in N-*ras* mutation. Maternal exposure to solvents and plastics during pregnancy raised the risk of K-*ras* mutation about three-fold and seven-fold, respectively, and maternal exposure to plastics after pregnancy was associated with an eight-fold higher risk. Maternal and paternal exposure to oil and coal products and other hydrocarbons before and during pregnancy was associated with about a two-fold greater risk of N-*ras* mutation.

In previous studies, parental occupational exposure to hydrocarbons (such as chlorinated solvents, benzene, and paints) has been linked to elevated childhood leukemia risk. The present study has extended these findings to include drugs of abuse and additional chemical exposures, and to link them to *ras* mutations. The authors conclude that parental exposures to hydrocarbons and mind-altering drugs, chemicals that have been previously suggested to increase the risk of childhood leukemia, are related to specific *ras* mutations in childhood ALL.

## Figures and Tables

**Figure:**
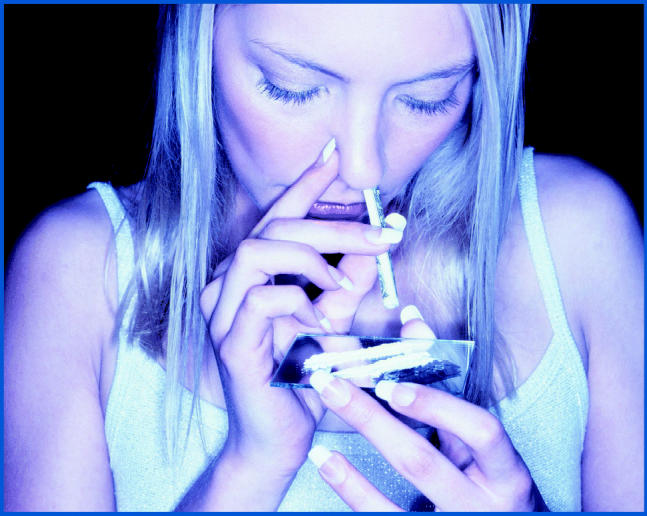
Shu XO, Perentesis JP, Wen W, Buckley JD, Boyle E, Ross JA, Robison LL; Children’s Oncology Group. 2004. Parental exposure to medications and hydrocarbons and *ras* mutations in children with acute lymphoblastic leukemia: a report from the Children’s Oncology Group. Cancer Epidemiol Biomarkers Prev 13(7):1230–1235.

